# Over 10-Year Outcomes of Infantile-Onset Epilepsies

**DOI:** 10.3390/jcm10030430

**Published:** 2021-01-22

**Authors:** Hyun-Jin Kim, Han Na Jang, Hyunji Ahn, Mi-Sun Yum, Tae-Sung Ko

**Affiliations:** 1 Department of Pediatrics, Myongji Hospital, Goyang 10475, Korea; rockhead8686@gmail.com; 2 Department of Pediatrics, Asan Medical Center Children’s Hospital, University of Ulsan College of Medicine, 88, Olympic-ro 43-gil, Songpa-gu, Seoul 05505, Korea; janghannah85@gmail.com (H.N.J.); network09@naver.com (H.A.)

**Keywords:** infantile-onset epilepsy, outcomes, prognostic factor

## Abstract

Seizures in infancy have highly variable courses and underlying etiologies. However, there are only a few long-term follow-up studies regarding infantile-onset epilepsy. Therefore, we aimed to describe the clinical courses, seizure outcomes, and risk factors of infantile-onset epilepsy followed up for more than 10 years in a tertiary center. Methods: Data of the patients with epilepsy, diagnosed under the age of 12 months and followed up for more than 10 years, were retrieved from the electronic medical records of Asan Medical Center Children’s Hospital. The patients’ medical records were retrospectively reviewed, and clinical outcomes were assessed based on the duration of seizure freedom at the last follow-up. Results: Of the 146 patients, 103 (70.5%) entered at least one remission, of whom epilepsy was resolved in 46 (31.5%). Forty-nine (33.6%) were found to be intractable at last contact. Delayed development, neurological deficits, and later onset (>3 months) were significantly associated with intractable epilepsies (*p* < 0.01). Conclusions: This study demonstrated that many patients with infantile-onset epilepsy can experience seizure remission. However, in some cases, early onset epilepsy was highly associated with various comorbidities and intractable seizures. Therefore, appropriate diagnosis and treatment are necessary to prevent further neuropsychiatric complications.

## 1. Introduction

Epilepsy is a common pediatric neurological disorder with numerous physical, psychosocial, and neurobehavioral morbidities, often resulting in a significant burden on the healthcare system and patients [1,2]. Childhood-onset epilepsy differs from epilepsy in adults not only in the clinical manifestations, including seizure type and epilepsy syndrome, but also in the presence of distinctive electroencephalogram (EEG) patterns, etiologies, and response to anti-seizure medication. The goals in treating epilepsy in children are complete control of their seizures, improvement of psychomotor development, and maintenance of quality of life for them and their family [3]. Previous follow-up studies [4,5,6,7] have reported that 60–70% of the patients with childhood-onset epilepsy entered remission under adequate treatment, and nearly half of them had sustained seizure remission following withdrawal of anti-seizure medication. In addition, some risk factors predicting poor prognosis have been identified, namely, symptomatic etiologies, epileptic encephalopathies, late response to medication, abnormal neuroimaging, any EEG slowing, and presence of developmental delay or intellectual disability.

The infancy period has the highest epilepsy incidence across all age groups, with epileptic spasms as the largest form of infantile epilepsy [8,9,10]. Seizures in infancy have variable courses, ranging from benign to severe [11], and a wide range of underlying etiologies, some of which require additional preventive management to avoid further neurodevelopmental damage, such as GLUT1 deficiency or aminoacidopathies [12]. Additionally, the first few years of life are especially critical for brain development, and poorly controlled seizures in this early period of life might cause severe cerebral impairment with various comorbidities [13,14,15,16,17]. To prevent further neuropsychiatric complications, therefore, early prediction of epilepsy outcome with proper management is crucial for all epilepsies diagnosed in infancy.

Apart from reports on epileptic spasms, however, there exist a limited number of long-term follow-up studies on the outcomes and prognosis of infantile-onset epilepsy [9,10,18,19,20]. Therefore, in the present study, we aimed to describe the clinical courses and outcomes of infantile-onset epilepsy in general and to evaluate the risk factors for intractability in a large cohort of patients followed up for more than 10 years in a tertiary center.

## 2. Materials and Methods

### 2.1. Study Design and Objectives

All case histories of children who were diagnosed with epilepsy and treated with routine anti-seizure medications at the Pediatric Neurology Department in Asan Medical Center were retrieved from electronic medical records. Patients with a single seizure or febrile seizures only and individuals with unclear clinical data were excluded. From 1995 to 2007, we identified 259 patients with a diagnosis of epilepsy before the age of 12 months. Among them, 146 patients followed up consecutively for more than 10 years at our center were included, and 113 patients were excluded due to inadequate medication (non-compliance or off medications despite seizures), loss of follow-up, uncertain outcomes, highly favorable course with short duration of follow-up (<10 years), or early death (7/259). Seizures were considered acute symptomatic if they occurred within the first 7 days of acute CNS insult, including structural, metabolic, toxic, infectious, or autoimmune causes [21]. The patients who experienced acute symptomatic seizures, as well as a single seizure or simple febrile seizures, were also excluded. However, some patients with acute symptomatic seizures followed by one or more unprovoked ones were diagnosed with epilepsy and were included in this study.

Patients’ medical records including sex, birth history, underlying conditions, previous history of status epilepticus, age at seizure onset, seizure semiology, and presence of neuropsychiatric deficits were reviewed. We also collected information on EEG, initial brain magnetic resonance imaging, genetic and metabolic findings, and history of anti-seizure medication use. Additionally, we searched the predictive factors for intractability, studying age at onset, sex, prematurity, history of hypoxic ischemic encephalopathy or status epilepticus (seizure lasting more than 30 min without fully regaining consciousness), epileptic spasms, neurologic deficit at diagnosis, presence of cognitive deficits or psychological problems, and neuroimaging findings as previously described [4,5,6,7,22]. This study is approved by Institutional Review Board of Asan Medical Center (IRB. No. 2018-0021).

### 2.2. Definitions

**Figure 1:**
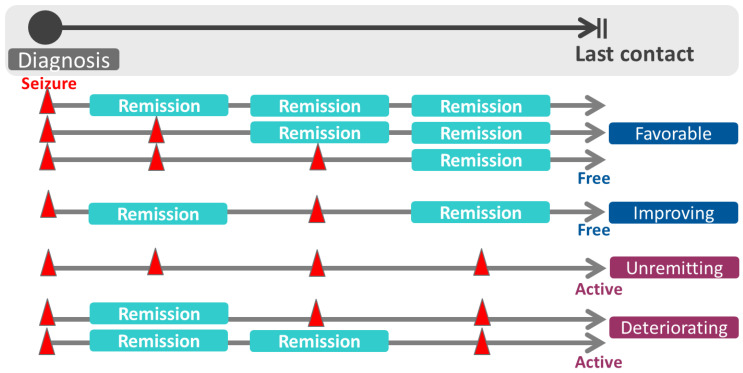
The course of epilepsy categorized by the remission assessed at 2 years, 5 years, and the last year of the follow-up period.

Classification of epilepsies and etiology of seizures were defined according to the new 2017 International League Against Epilepsy (ILAE) recommendations [23,24]. Etiology was classified into structural, genetic, metabolic, infectious, and unknown categories; patients were assigned to the etiologic category that was considered to be the most likely explanation for their epilepsy. Clinical course and outcome were assessed based on the duration of seizure freedom at the last follow-up. Remission of epilepsy was defined as a seizure-free period of 2 or more consecutive years regardless of anti-seizure medication use. At the end of follow-up, 5-year or longer periods of remission with or without use of anti-seizure medications was termed as terminal remission (TR). Resolution of epilepsy was defined as remaining seizure free for at least 10 years, with no seizure medications taken for the last 5 years [25]. Epilepsy was designated as intractable if remission was not achieved with adequate trials of two tolerated and appropriately chosen anti-seizure medications. Any subsequent seizure following remission was defined as relapse. The course of epilepsy was categorized based on the remission assessed at 2 years, 5 years, and the last year of the follow-up period. Various types of clinical courses are depicted in Figure 1 and defined as the following: a favorable course, as uninterrupted remission from the start of treatment to the last contact; an improving course, as remission detected at the time of final evaluation despite relapse during the follow-up period; an unremitting course with no remission at all assessment time points of follow-up; a deteriorating course, as early remission was interrupted by subsequent relapse and active seizures in the last contact [4]. To identify the proportion of the patients who have shown better outcomes and the associated factors, we divided the entire patient sample into good and bad course groups. Epilepsy with favorable and improving courses were defined as a relatively good course, while a poor course of epilepsy was defined as having unremitting and deteriorating courses. The possible factors associated with each good or poor course were further investigated.

### 2.3. Statistical Analyses

All collected data were analyzed using the Statistical Package for the Social Sciences (SPSS) version 18.0 (SPSS Inc., Chicago, IL, USA). The patients’ characteristics were analyzed concerning the demographic and clinical features using the chi-squared test. In order to evaluate several factors known to have associations with a prognosis of epilepsy, we initially used a chi-square test to determine whether there was a statistically significant difference between the categorical variables. The factors with high univariate significance (*p*-value < 0.05) were then subjected to a binary logistic regression analysis using the forward LR method to predict the adjusted odds ratio (OR) of being intractable or resolved using the values of the independent variables. The variables with *p*-value < 0.05 were defined to have statistical significance.

## 3. Results

One hundred forty-six infants who were followed up for more than 10 years were included in the study, and a retrospective review of their hospital charts was performed. The median age at epilepsy onset was 5.5 months, and the mean follow-up duration was 14.9 years (range, 10–23 years). With a slight male predominance (75/146, 51.4%), 21 (14.4%) patients were premature, 22 (15.1%) experienced hypoxic ischemic encephalopathy, and one had a history of congenital cytomegalovirus infection. Status epilepticus was observed in 8 (5.5%), and infantile spasm was identified as the most frequent type of seizure in 49 (33.6%) patients. Lennox–Gastaut syndrome was confirmed in 22 (15.1%) patients, of whom 13 (8.9%) evolved from West syndrome.

Forty-nine patients (33.6%) had underlying diseases, of which neurocutaneous syndromes, including tuberous sclerosis complex, neurofibromatosis type 1, Sturge–Weber syndrome, or incontinentia pigmenti, were the most common type of diseases in 15 (10.3%) patients. Metabolic diseases were observed in nine (6.2%) patients: three with citrullinemia, two with Leigh disease, one with ornithine transcarbamylase deficiency, one with pyridoxine dependent epilepsy, one with fumarase deficiency, and one with glutamate dehydrogenase 1 deficiency. Genetic diseases were observed in nine (6.2%) patients: eight with chromosomal abnormalities, including *1p36*, *18q22*, and *22q11.2* deletions and Down syndrome, and one with Noonan syndrome. Nine patients (6.2%) had tumors (three with infantile ganglioma, two with hypothalamic hamartoma, two with choroid plexus carcinoma, one with medulloblastoma, and one with retinoblastoma), and seven (4.8%) patients had hematologic or vascular diseases.

According to the new proposal for Epilepsy Classification (ILAE, 2017), most of the cases were classified into having structural (66/146, 45.2%), genetic (9/146, 6.2%), infectious (9/146, 6.2%), and metabolic causes (9/146, 6.2%). Regarding structural etiology, the most frequent findings were brain atrophy and/or encephalomalacia: 45 of the 146 (30.8%) patients. The remaining 53 (36.3%) were categorized into unknown etiology. Fifty-eight patients (39.7%) showed abnormal findings in EEG at least once during their follow-up: focal or multi-focal abnormalities in 48 (32.9%), abnormal background activities in 33 (22.6%), and generalized abnormalities in 5 (3.4%).

Co-morbid neurological conditions were identified in 80.8% (118/146); developmental delay or intellectual disability (106/146, 72.6%) was identified as the most common co-morbidity. Details of underlying diseases, etiologies, and comorbidities are presented in Table 1.

### 3.1. Clinical Courses and Epilepsy Outcomes

**Figure 2:**
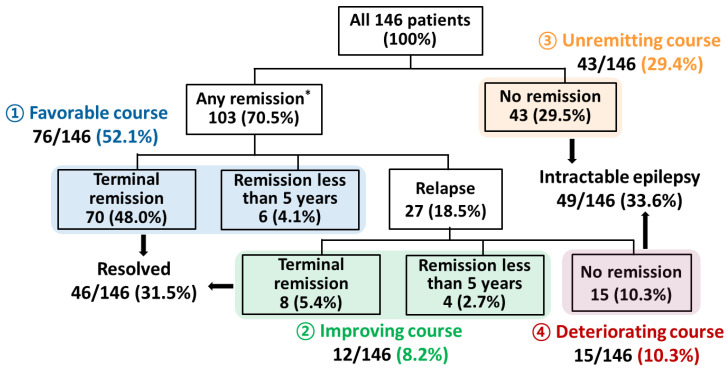
Long-term outcomes of the patients with infantile-onset epilepsy. * First remission (seizure free > 2 years) from the diagnosis.

Figure 2 shows the patients’ clinical courses and final outcomes. During the follow-up period, 103 out of 146 (70.5%) entered at least one remission period, while 43 (29.5%) had an unremitting course without any remission. Of the 103 patients with at least one remission, 27 (18.5% of total) experienced a subsequent relapse, and 15 of those (10.3% of total) had a deteriorating course with active epilepsy at last follow-up. Out of 58 patients who had unremitting or deteriorating courses, 49 (33.6% of total) were found to be intractable, while the remaining 9 (6.1% of total) had ongoing epilepsy that was more easily treated.

In contrast, most patients (88/146, 60.3%) achieved relatively good outcomes including a favorable course with uninterrupted remission in 52.1% (76/146) and an improving course with relapse followed by remission in 8.2% (12/146). Terminal remission was achieved in 70 patients with a favorable course and 8 with an improving course (78/146, 53.4% of total), of whom 46 (31.5% of total) ultimately met criteria for resolution of epilepsy at the end of the study.

### 3.2. Treatment

All 146 patients received anti-seizure medication treatment, and the mean duration of therapy was 10.5 years (median, 11.4 years; range, 0.4–21.1 years) with a median of 3.0 medications (range, 1–11). Sixty-eight (46.6%) patients who never achieved TR used more medications with a median of 5.5 years compared with that of the patients in TR (median, 2.0 years). Additionally, 118 (80.8%) patients had a history of polypharmacy (using two or more drugs) during the follow-up period. In all patients (146), phenobarbital was the most frequent medication, administered in 93 (63.7%) patients, with vigabatrin in 66 (45.2%) patients and valproic acid in 61 (41.8%) patients. In addition to anti-seizure medication treatment, 18.5% (27/146) of the patients underwent palliative therapies for intractable epilepsy. Ketogenic diet and vagal nerve stimulation (VNS) were administered in 18 (12.3%) and 7 (4.8%) patients, respectively. Fourteen (9.6%) patients received surgery, only two (2/14, 14.3%) of whom finally achieved TR.

### 3.3. The Predictive Factors for Epilepsy Outcomes

The following factors were analyzed to identify any associations with epilepsy outcomes at the end of follow-up. On univariate analysis, there were no statistically significant differences between the intractable and non-intractable groups regarding sex, prematurity, history of hypoxic ischemic encephalopathy or status epilepticus, psychosocial comorbidities, and neuro-radiologic findings (Table 2). However, the factors including age at seizure onset after 3 months (*p* = 0.002), developmental delay/intellectual disability (*p* < 0.001), neurologic deficits at diagnosis (*p* = 0.004), and infantile spasms (*p* = 0.015) were significantly associated with intractability at last contact. The factors with high univariate significance were then entered into the logistic regression model. Multivariable logistic regression analysis showed that the following factors significantly predicted a higher probability of intractability: (a) age at seizure onset after 3 months (OR 4.29, 95% confidence interval (CI) 1.69–10.86, *p* = 0.002), (b) developmental delay/intellectual disability (OR 4.26, 95% CI 1.30–13.96, *p* = 0.017), and (c) neurologic deficits at diagnosis (OR 2.57, 95% CI 1.11–5.96, *p* = 0.027). In contrast, normal cognitive development (OR 15.53, 95% CI 5.98–40.32, *p* < 0.001) and neuro-radiological findings (OR 3.70, 95% CI 1.45–9.49, *p* = 0.006) significantly increased the chance of achieving resolution at the end of study. Normal cognitive development (OR 10.52, 95% CI 3.46–31.99, *p* < 0.001) also predicted a higher chance of a favorable or improving course.

## 4. Discussion

We included 146 infants who were diagnosed with epilepsy within the first year of their life and followed up for more than 10 years in a tertiary center. Using this long-term follow-up data, we investigated the clinical course of epilepsy and associated risk factors of intractability in infantile-onset epilepsy.

The proportion of identified etiologies was higher (93/146, 63.7%) in our sample compared to what was previously observed in a retrospective Italian study for infantile epilepsy diagnosed within the first 3 years of life (54.9%) [5] but similar to what has been reported from Thailand for epilepsy with onset before the first year of life [19]. The majority of our patients (66/146, 45.2%) showed structural brain abnormalities. Genetic and metabolic etiologies were found to be similar at 6.2% (9/146). As exhaustive genetic investigations were not routinely performed for diagnosis of epilepsy at our center in the past, however, it might be possible that there were more hidden genetic or metabolic causes among the unknown etiology group. In this genomic era, advanced genetic techniques such as next-generation sequencing (NGS) or chromosomal microarray can be utilized in cases with previously unknown etiology and uncontrolled seizures to predict their prognosis and enable targeted therapy depending on the genetic mutation detected [26,27].

In this study, over 70% of patients (103/146) with infantile-onset epilepsy entered a remission period, and more than half of the patients (78/146) achieved TR at the last follow-up. Moreover, 60.3% (88/146) of patients showed a relatively good course of epilepsy. When infants are diagnosed with epilepsy, the parents are usually shocked and feel miserable, and these fears and frustrations do not help in the treatment of patients. To date, most research on infantile epilepsy has addressed infant spasms and specific genetic epilepsy with poor outcomes, and several comparable studies have also focused primarily on clinical characteristics or included later onset (>1 year) with a shorter follow-up period (a 2-year follow-up or more than a 1-year follow-up). Thus, these 10-year long-term follow-up data, showing that infantile epilepsy can disappear in teenage years or that the clinical course is expected to improve gradually, encourage parents with young children to actively engage in treatment.

However, 80.8% (118/146) of patients received polypharmacy, with one-third (49/146, 33.6%) of these patients having intractable epilepsy. Furthermore, relapse was observed in 26.2% (27/103) of the patients, who entered at least one remission. The proportion of intractable epilepsy in our study is higher than the estimates reported by previous works regarding epilepsy [4,28,29,30]. This difference can be mainly attributed to the early seizure onset and the bias of enrolment of patients with more severe epilepsy or with serious etiologies.

To identify the risk factors predictive of outcomes in patients with infantile-onset epilepsy with significant morbidity, we divided patients into two groups according to their seizure intractability. Three risk factors were identified on logistic regression analysis: age at seizure onset after 3 months, neurologic deficits at diagnosis, and developmental delay/intellectual disability, the latter being the main factor, with a nearly 4–5-fold higher risk of remaining intractable at the final evaluation. The result is consistent with those of previous studies [4,5,6,7] that have observed poorer outcomes in children with concomitant neurologic handicaps or intellectual disability, focal abnormalities in EEG, and symptomatic epilepsy. In contrast, younger age (<3 months) at seizure onset had relatively good outcomes. This is probably due to a low frequency of severe developmental and epileptic encephalopathies, such as West syndrome, in this age group, as well as a high incidence of subsequent unprovoked seizures, following acute symptomatic events with remediable causes [11,21,31]. However, we found no statistical differences between the intractable and non-intractable groups regarding etiologic diagnosis, which could be due to the heterogeneous severity of diseases involved in each structural, genetic, or unknown etiologies according to the revised classification of the ILAE Commission on Classification and Terminology, 2017 [23]. In addition, the presence of infantile spasms was a significant risk factor on univariate but not on multivariate analysis. This suggests that the age of onset, developmental delay/intellectual disability during follow-up, and neurological deficits at the time of diagnosis in children with infantile spasms are also risk factors for epilepsy intractability, rather than the presence of infantile spasms itself.

Recently, many studies have supported that epilepsy surgery at a younger age is effective for medically intractable epilepsy [32,33,34,35,36]. Kadish et al. [37] demonstrated that early epilepsy surgery is safe and efficient regarding long-term seizure freedom and anti-seizure medication cessation in selected patients and epilepsy duration is the only modifiable developmental predictor. Unfortunately, all 14 infants with epilepsy surgery in this cohort were treated with a palliative option, such as VNS or callosotomy. As we could not follow those who achieved complete seizure freedom at an early age for 10 or more years, it would be difficult to evaluate the effectiveness of curative epilepsy surgery in this study. However, early curative surgery can be seen as the method of choice for intractable focal epilepsy in children with infantile-onset epilepsy [38].

The major strength of our work is that this is a single center-based long-term follow-up study that demonstrates the outcomes of epilepsy with an onset specifically in the first year of life. Additionally, our cases represent a relatively uniform group of epilepsy patients, and all data collected during the entire follow-up are more reliable than those of earlier studies [9,19,20], since we performed designated diagnostic procedures and provided constant life-long management from diagnosis to the last follow-up. The retrospective design, possible biases towards more severe epilepsy, and high number of the excluded patients including some benign cases followed up for a short period of time, such as benign familial neonatal seizures, are the major limitations of the present study. Long-term prospective follow-up studies on those patients diagnosed within the last 10 years, who were excluded in our study, can further delineate the outcome of those with short-term follow-up period.

## 5. Conclusions

The results of our retrospective, tertiary center-based long-term follow-up study demonstrated that almost two-thirds of the infants with seizure-onset epilepsy within the first year of life can be well-controlled at last contact. However, early-onset seizures were highly associated with various comorbidities. Those with risk factors, such as age at seizure onset after 3 months, neurological deficits at diagnosis, and developmental delay/intellectual disability, had a significant risk of intractability. Therefore, appropriate diagnosis and treatment, with regular neurodevelopmental screenings, is necessary for all infantile-onset epilepsy to prevent further neuropsychiatric complications. Moreover, broad application of advanced genetic technologies would be helpful in providing proper targeted therapy and predicting prognosis in this genomic era.

## Figures and Tables

**Table 1:** Characteristics of 146 patients with infantile-onset epilepsy.

Male	75 (51.4%)
Prematurity	21 (14.4%)
Hypoxic ischemic encephalopathy	22 (15.1%)
Status epilepticus	8 (5.5%)
Infantile spasms	49 (33.6%)
Underlying disease	
Neurocutaneous syndromes	15 (10.3%)
Genetic diseases	9 (6.2%)
Metabolic diseases	9 (6.2%)
Tumors	9 (6.2%)
Hematologic or vascular diseases	7 (4.8%)
Etiology	
Structural	66 (45.2%)
Unknown	53 (36.3%)
Genetic	9 (6.2%)
Infectious	9 (6.2%)
Metabolic	9 (6.2%)
Abnormal findings in EEG	
Focal or multi-focal abnormalities	48 (32.9%)
Abnormal background activities ^1^	33 (22.6%)
Generalized abnormalities	5 (3.4%)
Comorbidities	
Developmental delay or intellectual disability	106 (72.6%)
Neurologic deficit at diagnosis	74 (50.7%)
Psychosocial problems ^2^	15 (10.3%)

^1^ Defined as non-physiological background electrical activities in EEG, including pathological synchrony, focal slow waves, and hypsarrhythmia. ^2^ Include ADHD, behavioral disorders, depression, and autism spectrum disorders.

**Table 2:** Univariate analysis for intractability at last contact.

	Non-Intractable (*N* = 97, 66.4%)	Intractable (*N* = 49, 33.6%)	Total (*N* = 146)	*p*-Value
Sex				0.18
Male	46	29	75	
Female	51	50	71	
Prematurity				0.60
≥GA 37 weeks ^1^	81	43	125	
<GA 37 weeks	15	6	21	
Hypoxic ischemic encephalopathy				0.01
No	79	45	124	
Yes	18	4	22	
Status epilepticus				0.08
No	94	44	138	
Yes	3	5	8	
Infantile spasms				0.015
No	71	26	97	
Yes	26	23	49	
Age at onset				0.002
≤3 months	41	8	49	
>3 months	56	41	97	
Etiology				0.07
Structural	41	25	66	
Unknown	37	16	53	
Genetic	6	3	9	
Infectious	7	2	9	
Metabolic	6	3	9	
Developmental delay or intellectual disability				<0.001
No	36	4	40	
Yes	61	45	106	
Neurologic deficit at diagnosis				0.004
No	56	16	72	
Yes	41	33	74	
Psychological problems				0.86
No	80	41	121	
Yes	17	8	25	

^1^ Gestational age of 37 weeks.

## Data Availability

The data presented in this study are available on request from corresponding authors.
